# Comparative Morphology of Wax Gland Heads in Adult Dustywings (Insecta: Neuroptera: Coniopterygidae)

**DOI:** 10.3390/insects14070650

**Published:** 2023-07-20

**Authors:** Min Li, John D. Oswald, Zhiqi Liu

**Affiliations:** 1 Department of Entomology, China Agricultural University, Beijing 100193, China; liminbetter@126.com; 2 Department of Entomology, Texas A&M University, College Station, TX 77843, USA

**Keywords:** Coniopterygidae, dustywings, wax production, exocrine glands, microtrichia, scanning electron microscopy, insect ultrastructure

## Abstract

**Simple Summary:**

Most of the body and wings of mature adults of most species of dustywings (Neuroptera: Coniopterygidae) are covered with a superficial coating of pale waxy particles. This white-to-gray waxy ‘dust’—from which the common name of the family derives—is secreted onto the outside of the body by wax glands that open on the head, thorax, and abdomen. Each gland opening produces a pair of tiny, curled wax filaments, which eventually break off as minute wax rings. The rings are subsequently distributed across most of the body and wing surfaces by the insect’s legs. Within the order Neuroptera, coniopterygids are unique in this form and use of waxy compounds, and it constitutes a distinctive shared feature of species in the subfamilies Aleurop-teryginae and Coniopteryginae (wax glands are apparently absent in the subfamily Brucheiserinae). Despite the interesting adaptive features that this system implies, wax production and its associated morphological features and behaviors are poorly known and rarely studied. In this paper, we examine the comparative morphology of wax gland head ultrastructure in 2 subfamilies, 9 genera, and 28 species of dustywings—the most diverse sample of dustywing taxa examined to date for this morphological feature. We report similarities and differences among the examined taxa and identify ultrastructural characters useful for taxonomic and phylogenetic differentiation at higher levels within the family.

**Abstract:**

In the largest comparative study of coniopterygid wax gland head morphology to date, we used scanning electron microscopy to illustrate the ultrastructure of gland heads found in 2 subfamilies (Aleuropteryginae and Coniopteryginae), 5 tribes (Aleuropterygini, Coniocompsini, Coniopterygini, Conwentziini, and Fontenelleini), 9 genera (*Aleuropteryx*, *Coniopteryx*, *Coniocompsa*, *Conwentzia*, *Cryptoscenea*, *Heteroconis*, *Semidalis*, *Spiloconis*, and *Thecosemidalis*), and 28 species of Palearctic and Oriental dustywings collected from a variety of sites across China. We propose a new descriptive terminology to concisely characterize the major elements of gland head ultrastructure and then identify similarities and differences among them and provide detailed descriptions of the wax gland heads found in each of the nine genera examined. Based on the range of taxa examined, we propose hypotheses about the functional morphology of some of the ultrastructural elements examined and relate them to wax ring formation in dustywings. An identification key for the examined genera based on gland head morphology is also presented.

## 1. Introduction

Many different groups of insects utilize exocrine glands to deposit waxy compounds onto the outer surfaces of their cuticles. This form of wax production has been studied in some detail in certain taxa of many insect orders, e.g., scale insects, aphids, planthoppers, psyllids, and whiteflies in the Hemiptera [1,2,3,4,5,6]; honey bee adults and sawfly larvae in the Hymenoptera [7,8]; some moth and butterfly larvae in the Lepidoptera [9,10]; ladybird larvae in the Coleoptera [11]; and dragonflies in the Odonata [12]. Across the Insecta, many different functions have been attributed to extracuticular waxes, including (among others): the reduction of water loss, resistance to wetting, the blockage of pathogen invasion, the avoidance of parasitism and/or predation, and the reflection of light of different wavelengths [3,11,12,13,14,15].

The family Coniopterygidae (Insecta: Neuroptera) is a group of small predaceous insects that feed principally on small arthropods such as scale insects, aphids, whiteflies, and mites. They can be used in biological control and are potentially important economic insects [16,17,18,19,20]. The family is worldwide in distribution and comprises ca. 600 extant species placed in 23 genera and 3 subfamilies: Brucheiserinae, Aleuropteryginae, and Coniopteryginae [21,22]. Brucheiserinae contains only four extant species and is known only from Argentina and Chile, while Aleuropteryginae and Coniopteryginae, which contain >99% of the species, are both distributed worldwide [23,24].

Adult coniopterygids are commonly called ‘dustywings’ because the bodies and wings of most species are covered with a superficial layer of pale, waxy, extracuticular particles. The waxes in this case are produced by glands that deposit curled wax filaments loosely onto the cuticle, which are then spread across the surface of the body by movement of the legs. The biological function of these particles is not well understood, but Gebhardt et al. [25] suggested—based on the complex shape, loose attachment, and strongly hydrophobic and anti-adhesive properties of the particles—that they may protect coniopterygids from entrapment in water and/or spiderwebs. In the Aleuropteryginae and Coniopteryginae, wax gland heads are known to occur on the head, thorax, and abdomen. On the abdomen, where they are particularly numerous, they typically occur in clusters or in transverse rows on the tergites and/or sternites [26].

The purpose of the present work is to expand our knowledge of wax gland head morphology by examining a broader range of coniopterygid taxa than have been studied previously. Here, we illustrate (using SEM), describe (using a newly proposed terminology), and compare the ultrastructure of exocrine wax gland heads found in 2 subfamilies (Aleuropteryginae, Coniopteryginae), 9 genera [five for the first time, *] (*Aleuropteryx*, *Coniopteryx*, Coniocompsa*, Conwentzia, Cryptoscenea*, Heteroconis*, Semidalis, Spiloconis*, and Thecosemidalis*), and 28 species of Palearctic and Oriental dustywings. We identify new characters and character states that may be useful in future taxonomic and phylogenetic studies of the Coniopterygidae and propose hypotheses about the possible functions of some of the novel structures found.

## 2. Materials and Methods

### 2.1. Species and Specimens Examined

The 28 species examined for this study are listed in Table 1 in taxonomic order. The collection data for the examined specimens is given in Table S1. The specimens were collected from a variety of locations across the Palearctic and Oriental biogeographic regions of China, mostly during the past five years. All specimens were field collected into 95% ethyl alcohol and are now deposited in the Entomological Museum, China Agricultural University, Beijing (CAU). The species were identified primarily using keys published by Meinander [26], Liu [27], Sziráki [21], and Zhao et al. [28]. Although we observed and imaged non-abdominal (i.e., cephalic and thoracic) wax gland heads for several species as part of the broader research for this study, here, we report only data from gland heads observed and imaged on the abdomen.

### 2.2. Scanning Electron Microscopy (SEM)

The body parts used for SEM examination were air-dried from 95% ethanol. For ‘whole body’ examinations, the wings were removed to expose the side of the body; for ‘abdomen’ examinations, the abdomens were removed from the rest of the body. The body parts were mounted on stubs using aluminum double-sided tape, coated with 15 nm of platinum using a high-vacuum sputter coater (LEICA EM ACE600) and then observed and imaged using a low-vacuum tabletop scanning electron microscope (Regulus 8100) at 10 kV.

Although we have not conducted comparative tests with other preservation, drying, and imaging techniques, it is likely that some of the techniques used here—particularly air-drying specimens from ethanol—have produced artifacts in the imaged surfaces that are not present in the cuticle of living or recently killed specimens. This pertains particularly to the development of irregular shrinkage ridges in the membranous cuticle surrounding each wax gland head, and the skewing of microtrichia and other structures borne on those surfaces. While most or all of the central region of the wax gland head appears to be sufficiently well sclerotized (in fully mature, non-teneral, specimens) to resist distortion from air drying effects, such effects are clearly present toward the outer margins of many of the gland heads imaged in this work, where the rigid parts of the head grade laterally into surrounding membranous tissue. These drying effects are individual to each specimen imaged and particularly affect the apparent shape, depth, and extent of the pella and possibly the orientations of structures borne on it (e.g., the wax guide microtrichia). As drying effects are expected to manifest especially in areas of weakly sclerotized cuticles, images of teneral specimens could display more widespread effects across the broader surface of a gland head. We are cognizant of these effects and took them into consideration in the development of our descriptions of gland head structures. The interpretations of gland head morphology contained in this work are based on the SEM images included herein, together with numerous other SEM images that were produced as part of the research for this work but are not published here.

## 3. Results

### 3.1. Overview of Wax Gland Head Structure

To facilitate the description and discussion of the primary ultrastructural features found in coniopterygid wax gland heads, we propose and define below a new terminology that we believe to be suitable for these purposes as no such terminology currently exists. We base most of the new terms on classical Greek and Latin words to encourage their international adoption. We use the phrase ‘wax gland head’ (short form: ‘gland head’) to encompass the morphological region centered on a wax gland foramen complex and extending laterally in all directions around this complex out to and including any other modifications of the cuticle that surround the foramen complex (e.g., modified microtrichia, ridges, clefts, depressions, and cuticular sculpturing) and which differentiate that cuticle from the ‘normal’ cuticle that surrounds the gland head (i.e., a membranous cuticle that displays no modifications that appear to be associated with the presence of a wax gland foramen). We assume here that the cuticle of the wax gland head region is sufficiently rigid (in fully mature, non-teneral, specimens) to resist distortion by drying (or other effects) out to its transition zone to the membranous cuticle at the outer margin of the pella. Thus, we assume (unless otherwise noted) that the microstructures displayed in the SEMs are accurate representations of wax gland head structure in mature adults. We use the term ‘opposite’ to refer to multiple structures that lie along (or nearly so, or across) one horizontal line drawn through the center point of the embola; the structures may lie along the line on the same side or on different sides of the embola. The term ‘alternate’ is used to refer to multiple structures that lie along (or nearly so, or across) two different horizontal lines drawn orthogonally through the center point of the embola.

#### 3.1.1. Coniopteryginae

**Figure 1:**
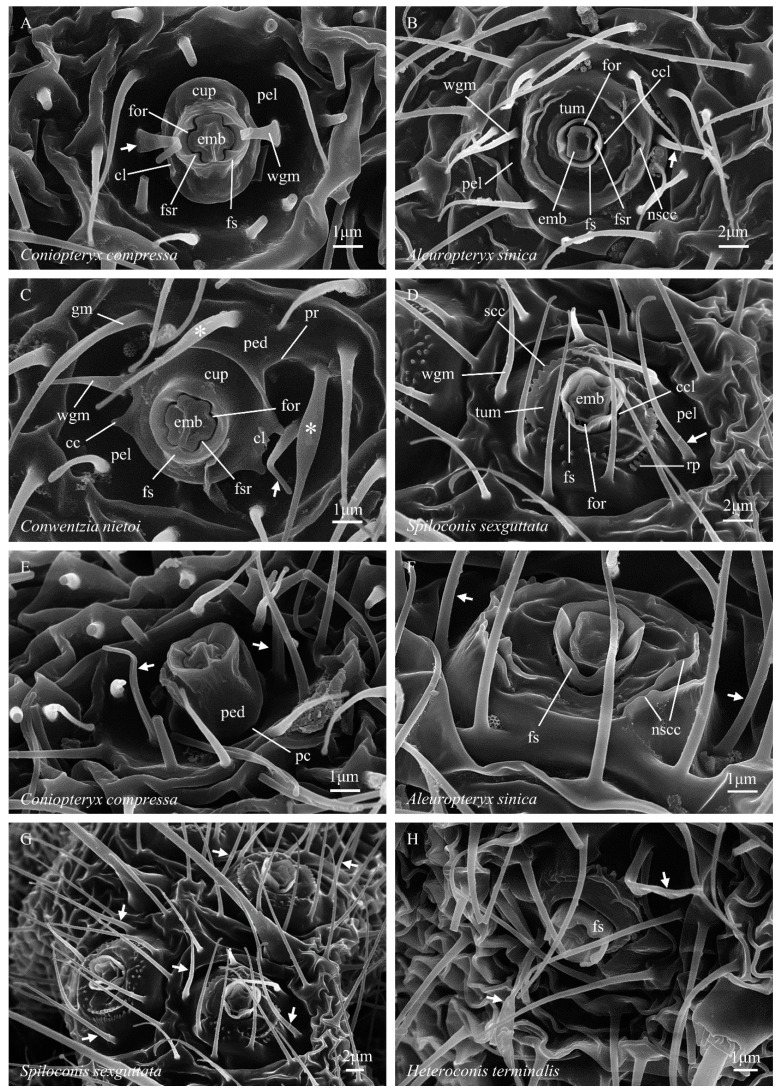
(**A**–**H**) Abdominal coniopterygid wax gland head terminology. Symbols and abbreviations: ↗—wax guide microtrichium; *—guard microtrichium; cc—cupular cusp; ccl—circular cleft; cl—cupular lobe; cup—cupula; emb—embola; for—foramen; fs—foramen sheath; fsr—foramen sheath ridge; gm (or *)—guard microtrichium; nscc—non-serrate concentric carina; pc—pedicelar constriction; ped—pedicel; pel—pella; pr—pedicellar ridge; rp—recumbent papilla; scc—serrate concentric carina; tum—tumulus; wgm (or ↗)—wax guide microtrichium.

**Figure 2:**
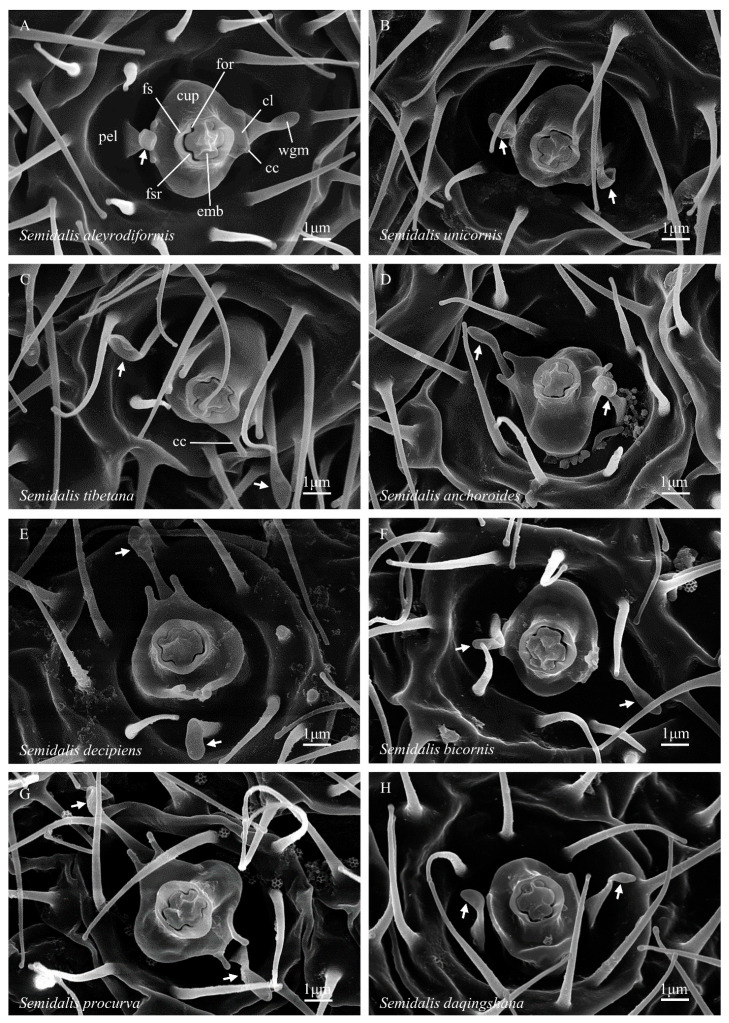
(**A**–**H**) Abdominal coniopterygid wax gland heads, Coniopteryginae: Conwentziini: *Semidalis* spp. Symbols and abbreviations: see Figure 1.

**Figure 3:**
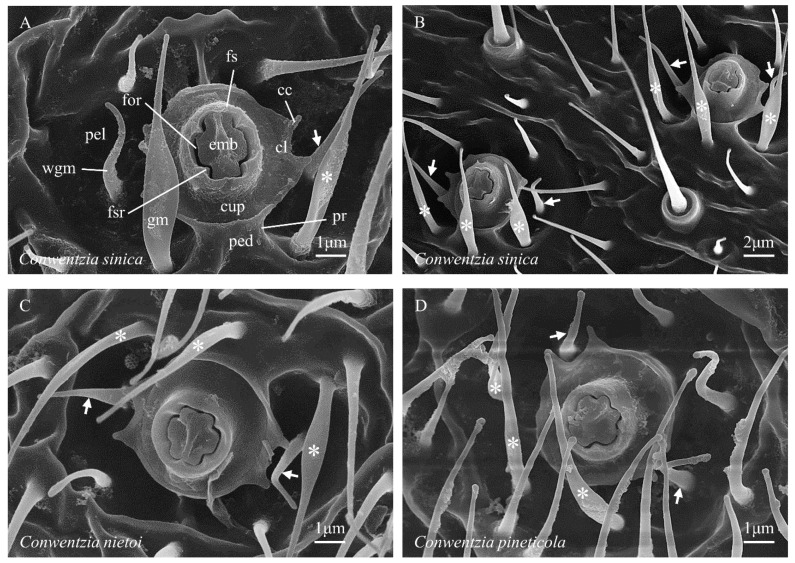
(**A**–**D**) Abdominal coniopterygid wax gland heads, Coniopteryginae: Conwentziini: *Conwentzia* spp. Symbols and abbreviations: see Figure 1.

**Figure 4:**
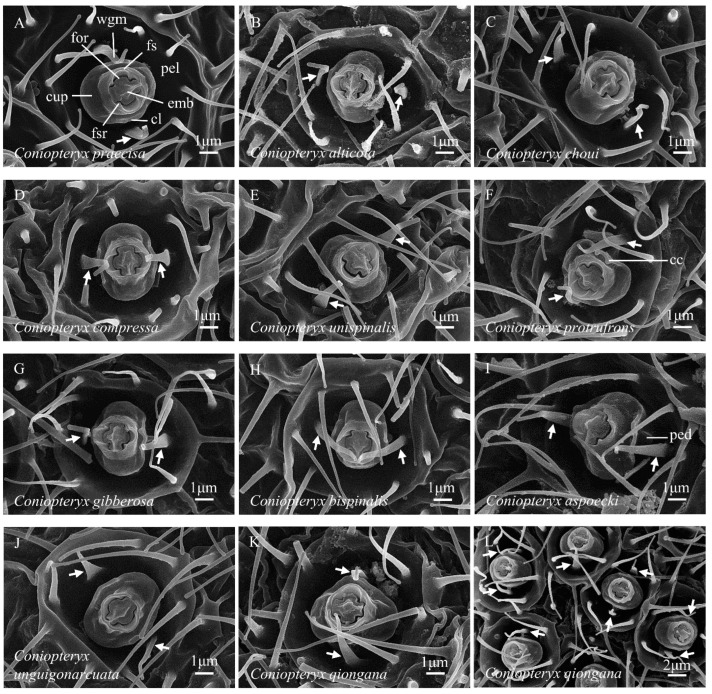
(**A**–**L**) Abdominal coniopterygid wax gland heads, Coniopteryginae: Coniopterygini: *Coniopteryx* spp. Symbols and abbreviations: see Figure 1.

**Figure 5:**
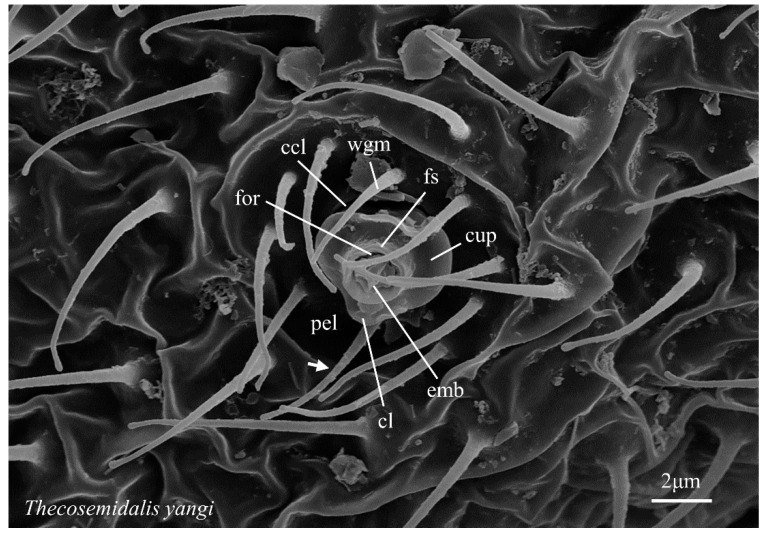
Abdominal coniopterygid wax gland head, Coniopteryginae: Coniopterygini: *Thecosemidalis yangi*. Symbols and abbreviations: see Figure 1.

In the examined species of Coniopteryginae, a typical gland head consists of the following primary substructures:

(1) The ‘foramen’ (from L. *foramen*, a hole or opening) of the wax gland duct is the narrow, irregularly annuloid space that forms the external opening of the wax gland duct, and through which secreted wax must pass to reach the outer surface of the insect’s cuticle (Figure 1A, for). In Coniopteryginae, the shape of the foramen is an annuloid in the form of a hollow cross with unequal but symmetrical arm lengths. The foramen is bounded to the inside by the cruciform outline (viewed from above) of the embola and to the outside by the more or less closely fitting four-ridged inner wall of the foramen sheath.

(2) The ‘embola’ (from Gr. *embolos*, a plug or wedge) is the morphological structure that partially blocks the center of the wax gland duct, diverting secreted wax compounds laterally as they exit the gland duct through the foramen (Figure 1A, emb). The precise nature of the attachment of the embola within the apex of the wax gland duct is currently unknown but could be studied by serial sectioning (e.g., see Nelson et al. [29], figure 5). It is currently unclear whether the embola is fixed in place (probably) or is capable of some degree of movement into and out of the end of the wax gland duct (less likely). The outer (lateral) margin of the embola forms the inner border of the foramen. In Coniopteryginae, the outline of the lateral margin of the embola (viewed from above) is broadly cruciform, with its major axis (i.e., the midline through its longer arms) and its minor axis (i.e., the midline through its shorter arms) being of different lengths. In Coniopteryginae, the long axis of the embola lies midway between, and perpendicular to, the axis of the two wax filaments that are extruded through the foramen, while the minor axis aligns with the midline of the extruded filaments. Parts of the outer margin of the embola shape the outer surfaces of the paired wax rings that form as soft wax is extruded past them.

(3) The ‘foramen sheath’ (from AS *sceath*, a case or cover; short form: ‘sheath’) is the morphological structure that surrounds the upper end of the wax gland duct and bears the embola (Figure 1A, fs). It takes the general form of a hollow cylinder through which the wax gland duct passes internally, and it surrounds and supports the embola. The inner face of the foramen sheath contains the outer border of the foramen. In Coniopteryginae, the foramen sheath is thick walled (relative to Aleuropteryginae) and projects to a level approximately even with or only slightly beyond (not well beyond) the outer surface of the embola, and its distal margin is weakly crenate to more or less truncate. The inner wall of the sheath bears four (not two, as in Aleuropteryginae) prominent longitudinal ridges (opposite the embola notches) (Figure 1A, fsr). Parts of the inner face of the foramen sheath shape the inner surfaces of the paired wax rings that form as soft wax is extruded past them.

(4) The phrase ‘foramen complex’ may be used to denote the combined foramen sheath + foramen + embola.

(5) The ‘cupula’ (from L. *cupula*, a small tub or vat; adj. = cupular) is a concave (viewed from above) lateral flaring that subtends and surrounds the base of the foramen complex (Figure 1A, cup). From above, the cupula is circular to oval in general outline, and its outer margin bears a pair of flattened upturned lobes (the ‘cupular lobes’, Figure 1A, cl) that arise opposite the short arms of the embola and the adjacent wax guide microtrichia. The distal margins of the cupular lobes may be ar a pair of cusps (the ‘cupular cusps’, Figure 1C, cc) or be simply rounded (Figure 5). The cupular cusps may be present as short acute processes (Figure 1C), as longer spinose processes (Figure 2G), or as simple obtuse angles (Figure 2H). When cupular cusps are present, they always consist of four cusps, grouped into two adjacent pairs, which lie on the margins of the cupular lobes on opposite sides of the cupula. The gaps between the paired adjacent cusps align with the minor axis of the embola (and, consequently, also with the long axes of the extruded wax filaments). This alignment suggests that the paired cupular cusps (when present) play a role in guiding the wax filaments during the process of their formation into rings (see Nelson et al. [29], figure 4A) and probably contribute to the highly regular form of the wax rings produced. The bottom surface of each cupular lobe is positioned in such a way that it blocks the free end of an extruding wax filament as it curves away from the foramen, then recurves back toward the central complex, and ultimately reaches a nearly 360° arc; this suggests that the cupular lobes may play a role (likely in conjunction with the cupular cusps and the wax guide microtrichia) in breaking the extruded wax filaments when they reach a nearly complete circle. The cupula is absent in examined Aleuropteryginae.

(6) The ‘pedicel’ (from L. *pedicellus*, a small slender stalk; adj. = pedicellar) is the columnar stalk below the cupula that supports the cupula + foramen complex and raises it above the floor of the pella (Figure 1E, ped). When the central complex is viewed at a steep angle from above, the pedicel is largely or entirely hidden from view by the laterally flared surface of the cupula. In *Conwentzia*, the pedicel bears a pair of longitudinal ridges (the ‘pedicellar ridges’, Figure 1C, pr) that arise from the pella, extend up the side of the pedicel, and then extend onto the ventral surface of the cupula. The ridges occur on only one side of the pedicel, with the long axis of the embola running between the two ridges (which are, thus, alternate to the cupular cusps and wax guide microtrichia). On their pellar ends, the ridges typically run between the bases of the three ‘guard microtrichia,’ which are also found in *Conwentzia* species. In adjacent gland heads, the pedicellar ridges appear to occur on the same sides of the gland heads (Figure 3B). In *Thecosemidalis* (Figure 5), but not in other examined Coniopteryginae, the base of the pedicel is surrounded by a deep circular cleft where the pedicel inserts into the pella. In other Coniopteryginae, the base of the pedicel may (Figure 1E, pc), or may not (Figure 1C, ped), be somewhat constricted at its junction with the pella, but it is not surrounded by a deep, narrow, cleft such that the pedicel appears to be inserted through the bottom of the pella.

(7) The phrase ‘central complex’ may be used to denote the combined foramen complex + cupula + pedicel.

(8) The ‘pella’ (from Gr. *pella*, a cup or bowl; adj. = pellar) is (in Coniopteryginae) the concave depression within which the central complex sits and from the base of which the central complex arises (Figure 1A, pel). The margins of the pella grade laterally into ‘normal’ cuticle. The apparent shape and depth of the pella in examined specimens may be influenced by desiccation effects and the rigidity of the cuticle in individual specimens. While largely glabrous, all examined Coniopteryginae possess a pair of distinctively located microtrichia (the ‘wax guide microtrichia’, Figure 1A, wgm) that arise from the floor of the pella opposite, and a small distance lateral to, the cupular lobes. The locations of these microtrichia align with the minor axis of the embola. The physical positions of these two microtrichia strongly suggest that they play roles in guiding the two wax filaments extruded from the foramen as they elongate and form into rings (see Nelson et al. [29], figure 4A). The detailed form of the wax guide microtrichia varies among coniopterygine genera. In *Conwentzia* and *Thecosemidalis* (Figure 1C, wgm) they are slender, attenuated, and acuminate, with a circular cross-section (i.e., little, if any, modified from the ‘normal’ microtrichia lying adjacent to the gland head). In *Coniopteryx* (Figure 1A, wgm), they are broadened at the base, flattened, and attenuated throughout (i.e., oval in the cross-section), tapering to a fine rounded point. In *Semidalis* (Figure 2A, wgm), they are spatulate, with a broad flattened base, a medial constriction, and a flat, ovoid, distal expansion. In addition, *Conwentzia* species possess a second group of three (occasionally two) distinctively located and modified microtrichia. These flank one side of the margin of the cupula (alternate to the cupular lobs) and arise from the pella adjacent to the pedicellar ridges. We refer to these microtrichia as ‘guard microtrichia’ (Figure 1C, gm) in reference to their origin near the outer edge of the cupula. The guard microtrichia arise from a circular base, flare into a flattened medial region, and then narrow to an acuminate distal region. The function, if any, of the guard microtrichia is currently unknown. Their origin and location place them away from the path of wax filament extension, so they do not appear to play a role in wax ring formation.

#### 3.1.2. Aleuropteryginae

In the examined species of Aleuropteryginae, a typical gland head consists of the following primary substructures:

(1) The foramen slit appears somewhat wider than in Coniopteryginae due to the more loosely fitted margins of the adjacent embola and inner wall of the foramen sheath (Figure 1B, for).

(2) The general outline of the embola is rectangularly quadrate (not cruciform) (Figure 1B, emb). In some taxa, e.g., *Spiloconis* (Figure 1D, emb), one end of the long axis (alternate to the foramen sheath inner wall ridges) is narrowed, resulting in an irregular hexagonal outline.

(3) The foramen sheath (Figure 1B, fs) is subcircular in outline (viewed from above) and forms a thin-walled (relative to Coniopteryginae) hollow cylinder around the foramen and embola. In Aleuropteryginae, the sheath typically projects well beyond the top surface of the embola, which thus appears to be set relatively deeply within the sheath. The inner wall of the sheath bears two (not four, as in Coniopteryginae) prominent longitudinal ridges (opposite the wax guide microtrichia; Figure 1B, fsr). The distal margin of the foramen sheath may be unilabiate (with one concave marginal excavation; *Aleuropteryx*; Figure 1F, fs), bilabiate (with two concave marginal excavations; *Cryptoscenea* and *Spiloconis*; Figure 1D, fs), or strongly crenate (with six concave excavations separating six rounded lobes; *Coniocompsa* and *Heteroconis*; Figure 1H, fs). The single marginal concavity in *Aleuropteryx* and the two marginal concavities in *Cryptoscenea* and *Spiloconis* lie alternate to the foramen sheath inner wall ridges. Two of the six concavities in *Coniocompsa* and *Heteroconis* lie alternate to the foramen sheath inner wall ridges, while the other four flank the rounded lobes that lie opposite the foramen sheath inner wall ridges.

(4) The foramen complex (foramen sheath + foramen + embola) is inset within, and distinctly separated from, the margin of the adjacent tumular platform by a deep circular cleft, which closely surrounds the base of the outer wall of the sheath (Figure 1B,D, ccl). The cupula and pedicel are absent.

(5) The ‘tumulus’ (from L. *tumulus*, a mound or hillock; adj. = tumular), or tumular platform, is the low disk-shaped mound within the center of which the foramen complex is set (Figure 1B,D, tum). The margins of the tumulus grade laterally into ‘normal’ cuticle. The elevation of the tumulus grades generally downward away from the foramen complex into a shallowly depressed circular ring that marks its approximate outer limit. The surface of the tumulus may be either largely glabrous or bear (in different taxa) a variety of different forms of cuticular sculpturing. Notable sculpture types include: (a) irregular fields of minute papillae, sometimes recumbent (‘recumbent papillae’, Figure 1D, rp), and sometimes arranged into irregular lines, and (b) serrate and non-serrate carinae, often formed as shorter or longer arched ridges that are partially concentric around the foramen complex (‘serrate concentric carinae’, Figure 1D, scc, and ‘non-serrate concentric carinae’, Figure 1B, nscc). Some sculptural elements are arranged symmetrically on opposite sides of the tumular platform (e.g., Figure 1B,D), and their locations clearly correlate with the positions of elements within the foramen complex (e.g., the orientation of the embola and the position of the foramen sheath ridges). As such, these sculptural elements lie in characteristic positions relative to the orientation of the extruded wax filaments. In the Aleuropteryginae, we use the term pella to refer to the shallowly depressed ring that (in at least some taxa, e.g., Figure 1B,D, pel) surrounds and delimits the outer margin of the tumulus. The aleuropterygine pella typically gives rise to several microtrichia. The wax guide microtrichia (Figure 1B,D, wgm) occupy characteristic positions opposite the foramen sheath ridges, as in Coniopteryginae. In some genera (e.g., *Coniocompsa* and *Heteroconis*, Figure 1H, wgm) these microtrichia are morphologically differentiated from ‘normal’ microtrichia, while in other genera (e.g., *Aleuropteryx*, Figure 1B, wgm), they are not.

#### 3.1.3. Subfamily Differences in Wax Gland Head Morphology

The primary differences in wax gland head morphology found between the coniopterygid subfamilies Aleuropteryginae and Coniopteryginae are summarized in Table 2. In the Aleuropteryginae, the outline of the embola (viewed from above) is generally more or less rectangularly quadrate and the pella is restricted to a circular depression surrounding the tumular platform; the pedicel and cupula are absent. In the Coniopteryginae, the outline of the embola is broadly cruciform and the foramen complex rises from the center of a laterally flared cupula, which is borne on a short pedicel set at the bottom of a deep concavity (the pella); the tumular platform is absent.

### 3.2. Wax Gland Head Descriptions by Genus

For definitions of morphological terms, see “*Overview of Wax Gland Head Structure*” above. The descriptions presented below are based strictly on abdominal wax gland heads (i.e., excluding gland heads on the head or thorax). Where multiple congeneric species have been examined, the descriptions incorporate observations on all examined species.

#### 3.2.1. Aleuropteryginae: Aleuropterygini

*Aleuropteryx* (Figure 1B)—*embola*: shallowly inset within the foramen sheath, outline rectangularly quadrate, side/margin two-notched (opposite foramen sheath ridges); *foramen sheath*: outline subcircular, relatively thin walled, inner face two-ridged (opposite the embola notches), distal margin extending slightly beyond the top of embola and notched with one distinct concavity (alternate to the sheath ridges); *cupula*: absent; *pedicel*: absent; *tumulus*: bearing one or more non-serrate carinae opposite each foramen sheath ridge; carinae irregular but arched and concentric around the foramen complex; carinae absent or obsolete alternate to the foramen sheath ridges; serrate carinae and recumbent papillae absent; *pella*: a depressed ring surrounding and delimiting the tumular platform; wax guide microtrichia present, two (opposite foramen sheath ridges), not morphologically differentiated from the ‘normal’ microtrichia; guard microtrichia absent.

**Figure 6:**
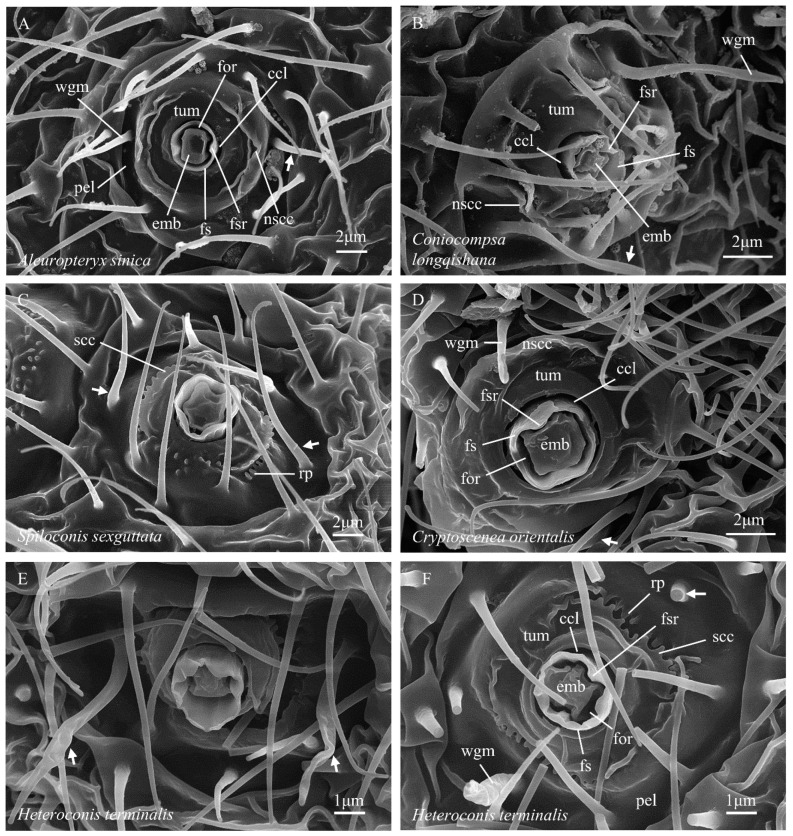
(**A**–**F**) Abdominal coniopterygid wax gland heads, Aleuropteryginae. Symbols and abbreviations: see Figure 1.

*Heteroconis* (Figure 6E,F)—*embola*: deeply inset within the foramen sheath, outline rectangularly quadrate, side/margin two-notched (opposite foramen sheath ridges); *foramen sheath*: outline subcircular, relatively thin walled, inner face two-ridged (opposite the embola notches), distal margin extending well beyond the top of the embola and strongly crenate (six-lobed); *cupula*: absent; *pedicel*: absent; *tumulus*: bearing one serrate carina opposite each foramen sheath ridge; carinae somewhat irregular but arched and concentric around the foramen complex; carinae absent alternate to the foramen sheath ridges, with this surface sometimes bearing short simple ridges; outer faces of serrate carinae flanked by a row of recumbent papillae; secondary arched carinae may be present between the serrate carinae and the foramen sheath; *pella*: a depressed ring surrounding and delimiting the tumular platform; wax guide microtrichia present, two (opposite foramen sheath ridges), morphologically differentiated from ‘normal’ microtrichia (base outline circular, thickened, and sometimes flattened distally); guard microtrichia absent.

#### 3.2.2. Aleuropteryginae: Coniocompsini

*Coniocompsa* (Figure 6B)—*embola*: deeply inset within the foramen sheath, outline rectangularly quadrate, side/margin two-notched (opposite foramen sheath ridges); *foramen sheath*: outline subcircular, relatively thin walled, inner face two-ridged (opposite embola notches), distal margin extending well beyond the top of the embola and strongly crenate (6-lobed); *cupula*: absent; *pedicel*: absent; *tumulus*: bearing one or more non-serrate carinae opposite each foramen sheath ridge; carinae irregular but arched and concentric around foramen complex; carinae absent or obsolete alternate to the foramen sheath ridges; serrate carinae and recumbent papillae absent; *pella*: a depressed ring surrounding and delimiting the tumular platform; wax guide microtrichia present, two (opposite foramen sheath ridges), morphologically differentiated from ‘normal’ microtrichia (base outline circular, flattened distally); guard microtrichia absent.

#### 3.2.3. Aleuropteryginae: Fontenelleini

*Cryptoscenea* (Figure 6D)—*embola*: shallowly inset within the foramen sheath, outline rectangularly quadrate, side/margin two-notched (opposite foramen sheath ridges); *foramen sheath*: outline subcircular, relatively thin walled, inner face two-ridged (opposite the embola notches), distal margin extending slightly beyond the top of the embola and notched with two distinct concavities (alternate to the sheath ridges); *cupula*: absent; *pedicel*: absent; *tumulus*: bearing one or more non-serrate carinae (opposite the sheath ridges); carinae irregular but arched and concentric around the foramen complex; carinae absent or obsolete alternate to the sheath ridges; serrate carinae and recumbent papillae absent; *pella*: a depressed ring surrounding and delimiting the tumular platform; wax guide microtrichia present, two (opposite foramen sheath ridges), not morphologically differentiated from ‘normal’ microtrichia; guard microtrichia absent.

*Spiloconis* (Figure 1D)—*embola*: deeply inset within the foramen sheath, outline rectangularly quadrate (or irregularly hexagonal), side/margin two-notched (opposite foramen sheath ridges); *foramen sheath*: outline subcircular, relatively thin walled, inner face two-ridged (opposite embola notches), distal margin extending well beyond the top of the embola and notched with two distinct concavities (alternate to the sheath ridges), bottom of one concavity sometimes bearing a small tooth (Figure 1D); *cupula*: absent; *pedicel*: absent; *tumulus*: bearing one serrate carina opposite each foramen sheath ridge; carinae somewhat irregular but arched and concentric around the foramen complex; carinae absent alternate to the foramen sheath ridges, with this surface bearing small upright papillae; outer faces of serrate carinae flanked by a row of recumbent papillae; *pella*: a depressed ring surrounding and delimiting the tumular platform; wax guide microtrichia present, two (opposite foramen sheath ridges) not morphologically differentiated from ‘normal’ microtrichia; guard microtrichia absent.

#### 3.2.4. Coniopteryginae: Coniopterygini

*Coniopteryx* (Figure 1A)—*embola*: shallowly inset within the foramen sheath, form broadly cruciform with two longer and two shorter arms, axis of long arms alternate to wax filament elongation, top of the long axis typically raised, outline four-notched (opposite foramen sheath ridges); *foramen sheath*: outline subcircular, relatively thick walled, inner face four-ridged (opposite embola notches), distal margin weakly crenate to truncate; *cupula*: present; outline distinctly oval (due to upturned cupular lobes); four cupular cusps present, with the cusps arranged in two pairs, one pair on margin of each cupular lobe, gap centers between the cusp pairs aligned with midline of the short arms of the embola, cusps angular to short protruding; *pedicel*: present, short, longitudinal ridges absent, deep circular cleft at the pedicel–pella junction absent; *pella*: a deep, more-or-less hemispherical depression; wax guide microtrichia present, two (opposite cupular cusp gaps), base broad and flattened, flattened and attenuate distally, often inflexed; guard microtrichia absent.

*Thecosemidalis* (Figure 5)—*embola*: shallowly inset within the foramen sheath, form broadly cruciform with two longer and two shorter arms, with the axis of the long arms alternate to wax filament elongation, top of the long axis raised, outline four-notched (opposite foramen sheath ridges); *foramen sheath*: outline subcircular, relatively thick walled, inner face four-ridged (opposite embola notches), distal margin weakly crenate to truncate; *cupula*: present; outline oval (due to upturned cupular lobes); cupular lobes rounded, lacking marginal cusps; *pedicel*: present, short, longitudinal ridges absent, base surrounded by a deep circular cleft at the insertion within the pella; *pella*: a deep, more-or-less hemispherical depression; wax guide microtrichia present, two (opposite cupular lobes), form not differentiated from ‘normal’ microtrichia; guard microtrichia absent.

#### 3.2.5. Coniopteryginae: Conwentziini

*Conwentzia* (Figure 1C)—*embola*: shallowly inset within the foramen sheath, form broadly cruciform with two longer and two shorter arms, axis of the long arms alternate to wax filament elongation, top of the long axis typically raised, outline four-notched (opposite the foramen sheath ridges); *foramen sheath*: outline subcircular, relatively thick walled, inner face four-ridged (opposite embola notches), distal margin weakly crenate to truncate; *cupula*: present; outline subcircular (due to laterally flared cupular lobes); four cupular cusps present, arranged in two pairs, one pair on the margin of each cupular lobe, the gap centers between the cusp pairs opposite the midline of the short arms of the embola, cusps protruded as short acute processes; *pedicel*: present, short, one face with two longitudinal ridges present, deep circular cleft at the pedicel–pella junction absent; *pella*: a moderately deep rounded depression; wax guide microtrichia present, two (opposite cupular cusp gaps), form not differentiated from ‘normal’ microtrichia; guard microtrichia present, three (occasionally two), arising from the floor of the pella adjacent to, and separated by, the pellar ends of pedicellar ridges, form: base circular, broadened and flattened medially, distally narrowed and acuminate.

*Semidalis* (Figure 2A)—*embola*: shallowly inset within the foramen sheath, form broadly cruciform with two longer and two shorter arms, axis of the long arms alternate to wax filament elongation, top of the long axis typically raised, outline four-notched (opposite foramen sheath ridges); *foramen sheath*: outline subcircular, relatively thick walled, inner face four-ridged (opposite embola notches), distal margin weakly crenate to truncate; *cupula*: present; outline oval to subcircular (depending on upturn angle of cupular lobes); four marginal cusps present, arranged in pairs, one pair on the margin of each cupular lobe, the gap centers between the cusp pairs opposite the midline of the short arms of the embola, cusps simply angular, or as short acute processes, or protruded as distinct, parallel-sided processes; *pedicel*: present, short, longitudinal ridges absent, deep circular cleft at the pedicel–pella junction absent; *pella*: a deep, more-or-less hemispherical depression; wax guide microtrichia present, two (opposite the cupular cusp gaps), spatulate, with a broad flattened base, a medial constriction, and a flat, ovoid, distal expansion; guard microtrichia absent.

### 3.3. Identification Key to Genera

To highlight the most easily observable differentiating traits found in dustywing abdominal wax gland heads we present below a preliminary key to the nine coniopterygid genera that have been documented for this character complex to date. The key is based primarily on work undertaken for the present paper, but also incorporates observations from other published illustrations of coniopterygid wax gland heads. Although multiple species have been examined for several genera—i.e., *Coniopteryx*, *Conwentzia*, and *Semidalis*—most genera currently documented for wax gland head morphology are still known from only a single species. The ‘genus level’ generality of all of the traits utilized in this preliminary key requires further verification across a broader range of taxa.

Key to genera of Coniopterygidae based on abdominal wax gland head traits(Adult males and females; genera with documented gland heads only)1*Cupula*: present (Figure 1A); *foramen sheath*: inner wall four-ridged (Figure 1A); *embola*: outline cruciform (Figure 1A)(Coniopteryginae) 21′*Cupula*: absent (Figure 1B); *foramen sheath*: inner wall two-ridged (Figure 1B); *embola*: outline not cruciform (Figure 1B)(Aleuropteryginae) 52(1)*Guard microtrichia*: absent (Figure 1A); *pedicel*: longitudinal ridges absent (Figure 1E)32′*Guard microtrichia*: present (Figure 1C); *pedicel*: longitudinal ridges present (Figure 1C)
*Conwentzia*
3(2)*Cupula*: cupular cusps present (Figure 1C); *wax guide microtrichia*: flattened, cross-section oval (Figure 1A)43′*Cupula*: cupular cusps absent (Figure 5); *wax guide microtrichia*: not flattened, cross-section circular (Figure 5)
*Thecosemidalis*
4(3)*Wax guide microtrichia*: evenly attenuate, apex acuminate (Figure 1A)
*Coniopteryx*
4′*Wax guide microtrichia*: spatulate, apex ovoid, flattened, and expanded beyond a mid-length constriction (Figure 2A)
*Semidalis*
5(1′) *Foramen sheath*: distal margin uni- or bilabiate (i.e., with either 1 [Figure 1F] or 2 [Figure 1D] distinct emarginations)65′*Foramen sheath*: distal margin crenate, generally 6-lobed (Figure 1H)86(5)*Foramen sheath*: distal margin bilabiate (i.e., with two distinct emarginations) (Figure 1D)76′*Foramen sheath*: distal margin unilabiate (i.e., with one distinct emargination) (Figure 1F)
*Aleuropteryx*
7(6)*Tumulus*: arched carinae non-serrate, recumbent papillae absent (Figure 6D)
*Cryptoscenea*
7′*Tumulus*: arched carinae serrate, recumbent papillae present (Figure 1D)
*Spiloconis*
8(5′)*Tumulus*: arched carinae non-serrate, recumbent papillae absent (Figure 6B)
*Coniocompsa*
8′*Tumulus*: arched carinae serrate, recumbent papillae present (Figure 6F)
*Heteroconis*


## 4. Discussion

### 4.1. General

The study of coniopterygid waxes, wax-producing structures, and wax production processes is still in its infancy. The study by Nelson et al. [29] contains the only detailed examination to date of the chemical composition of a coniopterygid wax. In Table 3, we summarize the literature known to us that contains text descriptions and/or illustrations of coniopterygid wax gland head structures and/or wax particles. Prior to the current work, descriptive data had accumulated on abdominal wax gland head structure for only four genera and six species (and on wax particles for four genera) but with no more than three species treated in any single work. The present work provides new data on gland head structure for an additional 5 genera and 27 species and is the first work to examine the detailed ultrastructure of wax gland heads in a substantial number of taxa (9 genera and 28 species), thus providing the first opportunity for a broader comparative assessment.

Although minor differences in gland head structure may exist in some congeneric species, the preparation techniques and the conspecific specimen sample sizes used in this work were insufficient (and not designed) to differentiate between intra- and interspecific variation in these traits. Within all three genera for which we examined multiple species (*Coniopteryx*, 11 spp.; *Conwentzia*, 3 spp.; and *Semidalis*, 8 spp.), all congeneric species exhibited very similar gland head structures, but differences were apparent among the genera (as noted above). This observation supports the hypothesis that gland head structure may be broadly conserved within coniopterygid genera as they are currently defined. But only 9 of the 23 extant coniopterygid genera (39%) have been documented to date for gland head morphology, and multiple species have been documented for only 4 of those genera (*Aleuropteryx*, *Coniopteryx*, *Conwentzia*, and *Semidalis*). Because of the observed similarity in gland head structure among the congeneric species, we focused the descriptive and comparative aspects of this work on the higher taxonomic levels of genus and subfamily. The new terminological and descriptive framework erected here for wax gland heads should facilitate the integration of additional taxa into future and more comprehensive surveys and assessments of this character complex.

### 4.2. Taxonomy and Phylogeny

As the descriptions and identification key presented above attest, wax gland heads comprise a state-rich character complex that may be of substantial taxonomic utility at the genus and subfamily levels within the Coniopterygidae. However, taxon sampling for this character suite is still low (39% of coniopterygid genera and <6% of the family’s species), so it is still unclear how well the identified characters and states will stand up to rigorous comparative examination of additional taxa. Many of the characters/states that are of taxonomic interest will also prove to be of phylogenetic interest. However, as the phylogeny of the Coniopterygidae is currently not well resolved, it is difficult to be certain about polarities among the states of many of the gland head characters identified here; so we resist making phylogenetic statements in this work.

Several outstanding questions that particularly merit future investigation in a more rigorous phylogenetic context include: (1) is the absence of wax glands in Brucheiserinae plesiomorphic or a derived secondary loss within the Coniopterygidae? (2) The central complex and tumular platform are dominant characteristics of the subfamilies Coniopteryginae and Aleuropteryginae, respectively; what are their precursors and how did they evolve? (3) Are the deep circular clefts that surround the bases of the aleuropterygine foramen complex and the *Thecosemidalis* pedicel homologous or analogous? If homologous, do they provide any insights into the origins of the central complex and tumular platform? (4) How do the individual elements of the wax gland head function together to produce the wax rings that are so characteristic of adult coniopterygids, and does that functionality vary among different lineages of dustywings?

## 5. Conclusions

The expanded array of coniopterygid taxa examined in this work clearly identifies wax gland heads as a state-rich character complex that contain traits of both taxonomic value and phylogenetic interest. Our ability to develop a preliminary genus-level key based solely on gland head traits demonstrates that many of the traits in this complex correlate with current generic and/or subfamilial divisions of the Coniopterygidae and suggests that the complex merits additional study as a potential source of additional novel traits of taxonomic value. The higher-level taxonomic value (i.e., genus and subfamily) of many of the identified gland head traits suggests that they may also be of considerable antiquity and of significant phylogenetic interest, but our ability to interpret the traits in a phylogenetic context is hampered by the relative immaturity of the phylogenetic studies currently available for the family. Our extended documentation of the diverse array of fixed elements within the wax gland head character complex provides an additional data set from which to begin the development of hypotheses concerning the formation of the highly distinctive wax rings produced by dustywings.

## Figures and Tables

**Table 1:** Examined species, sexes, and body parts.

Subfamily: Tribe *Genus (Subgenus)* *Species*	Sexes	Body Parts
**Aleuropteryginae: Aleuropterygini**		
*Aleuropteryx sinica* Liu & Yang, 2003	1♂, 1♀	whole body
*Heteroconis terminalis* Banks, 1913	2♂	whole body
**Aleuropteryginae: Coniocompsini**		
*Coniocompsa longqishana* Yang & Liu, 1993	1♀	abdomen
**Aleuropteryginae: Fontenelleini**		
*Cryptoscenea orientalis* Yang & Liu, 1993	2♂, 1♀	whole body
*Spiloconis sexguttata* Enderlein, 1907	1♂, 1♀	whole body
**Coniopteryginae: Coniopterygini**		
***Coniopteryx (Coniopteryx)***		
*Coniopteryx alticola* Sziráki, 2002	1♂	abdomen
*Coniopteryx aspoecki* Kis, 1967	1♂, 1♀	whole body
*Coniopteryx bispinalis* Liu & Yang, 1993	1♂	abdomen
*Coniopteryx choui* Liu & Yang, 1998	1♂	abdomen
*Coniopteryx compressa* Yang & Liu, 1999	1♂	abdomen
*Coniopteryx gibberosa* Yang & Liu, 1994	1♂	abdomen
*Coniopteryx praecisa* Yang & Liu, 1994	1♂	abdomen
*Coniopteryx protrufrons* Yang & Liu, 1999	1♂	abdomen
*Coniopteryx unispinalis* Liu & Yang, 1994	1♂	abdomen
***Coniopteryx (Xeroconiopteryx)***		
*Coniopteryx qiongana* Liu & Yang, 2002	1♂	abdomen
*Coniopteryx unguigonarcuata* Aspöck & Aspöck, 1968	1♂	abdomen
*Thecosemidalis yangi* Liu, 1995	1♂	whole body
**Coniopteryginae: Conwentziini**		
*Conwentzia nietoi* Monserrat, 1982	1♂	abdomen
*Conwentzia pineticola* Enderlein, 1905	1♂	abdomen
*Conwentzia sinica* Yang, 1974	1♂, 1♀	whole body
*Semidalis aleyrodiformis* Stephens, 1836	1♂, 1♀	whole body
*Semidalis anchoroides* Liu & Yang, 1993	1♂	abdomen
*Semidalis bicornis* Liu & Yang, 1993	1♂	abdomen
*Semidalis daqingshana* Liu & Yang, 1994	1♂	abdomen
*Semidalis decipiens* Roepke, 1916	1♂	abdomen
*Semidalis procurva* Zhao et al., 2021	1♂	abdomen
*Semidalis tibetana* Zhao et al., 2021	1♂	abdomen
*Semidalis unicornis* Meinander, 1972	1♂	abdomen

**Table 2:** Primary differences in wax gland head morphology between the Aleuropteryginae and Coniopteryginae.

Coniopterygidae (Subfamily)	Embola (Outline)	Tumular Platform	Cupula	Pedicel
Aleuropteryginae	+/− rectangularly quadrate	present	absent	absent
Coniopteryginae	cruciform	absent	present	present

**Table 3:** Coniopterygid taxa for which descriptions (descr.) and/or detailed illustrations (illustr.) of wax gland heads and/or wax particles are currently available. ‘Figure’ (capital ‘F’) is used to refer to figures published in the present work; ‘figure’ (lower-case ‘f’) refers to figures published in other works. Lower-case letters used in the body of the table (e.g., a, b, c) refer to the corresponding letters given in the Citation column for the same row.

Taxon	Gland Head		Wax Rings		Citation
	Descr.	Illustr.	Descr.	Illustr.	
Aleuropteryginae sp. (no further identification)	−	−	a	b	[25]: 20−22 [a]; [30]; [31]: figure 1, right [b]
*Aleuropteryx* (genus)	+	−	−	−	present work
*Aleuropteryx* sp.	a	b	−	−	[32]: 9 [a], figure 2d [b]
*Aleuropteryx juniperi*	a	b	c	−	[33]: 655 [a], figure 1k [b]; [34]: 110 & figure 4 [c]
*Aleuropteryx sinica*	+	Figure 6A	−	−	present work
*Heteroconis terminalis*	+	Figure 6F	−	−	present work
*Coniocompsa longqishana*	+	Figure 6B	−	−	present work
*Cryptoscenea orientalis*	+	Figure 6D	−	−	present work
*Spiloconis sexguttata*	+	Figure 6C	−	−	present work
*Neoconis* sp.	−	−	−	a	[35]: figure 4 [a]
Coniopteryginae sp. (no further identification)	−	−	a	b	[25]: 20−22 [a]; [30]; [31]: figure 1, left [b]
*Coniopteryx* (genus)	+	−	−	−	present work
*Coniopteryx alticola*	−	Figure 4B	−	−	present work
*Coniopteryx aspoecki*	−	Figure 4I	−	−	present work
*Coniopteryx bispinalis*	−	Figure 4H	−	−	present work
*Coniopteryx choui*	−	Figure 4C	−	−	present work
*Coniopteryx compressa*	−	Figure 4D	−	−	present work
*Coniopteryx gibberosa*	−	Figure 4G	−	−	present work
*Coniopteryx haematica*	a	b	a	b	[36]: 271−272 [a], figures 20, 21 and 23 [b]
*Coniopteryx praecisa*	−	Figure 4A	−	−	present work
*Coniopteryx protrufrons*	−	Figure 4F	−	−	present work
*Coniopteryx pygmaea*	a	b, c, e	−	d, f	[31]: figure 3a,b [e], e,f [f]; [34]: figures 1 and 2 [d]; [37]: 313 [a], figure 3k [b], figure 7e,f [c]
*Coniopteryx unispinalis*	−	Figure 4E	−	−	present work
*Coniopteryx qiongana*	−	Figure 4K	−	−	present work
*Coniopteryx unguigonarcuata*	−	Figure 4J	−	−	present work
*Thecosemidalis yangi*	+	Figure 5	−	−	present work
*Conwentzia* (genus)	+	−	−	−	present work
*Conwentzia nietoi*	−	Figure 3C	−	−	present work
*Conwentzia pineticola*	−	Figure 3D	−	−	present work
*Conwentzia psociformis*	c	d, e	a, c	b, d	[31]: figure 3c,d [e]; [36]: 272–273 [c], figure 29 [d]; [38]:184 [a], pl. 8, figures 38 and 39 [b]
*Conwentzia sinica*	−	Figure 3A	−	−	present work
*Semidalis* (genus)	+	−	−	−	present work
*Semidalis* sp.	−	−	−	a	[35]: figure 3 [a]
*Semidalis aleyrodiformis*	a	b, Figure 2A	−	c	[33]: 655 [a], figure 2j,l [b], k [c]; present work
*Semidalis anchoroides*	−	Figure 2D	−	−	present work
*Semidalis bicornis*	−	Figure 2F	−	−	present work
*Semidalis daqingshana*	−	Figure 2H	−	−	present work
*Semidalis decipiens*	−	Figure 2E	−	−	present work
*Semidalis flinti*	a	b, d	a	c, d	[29]: 345−346 [a], figures 3a,b, 4a and 5 [b], figure 4b [c]; [31]: figure 2 [d]
*Semidalis procurva*	−	Figure 2G	−	−	present work
*Semidalis tibetana*	−	Figure 2C	−	−	present work
*Semidalis unicornis*	−	Figure 2B	−	−	present work

## Data Availability

All data are provided in the manuscript and the supporting documents.

## Supplementary Materials

The following supporting information can be downloaded at: https://www.mdpi.com/article/10.3390/insects14070650/s1. Table S1: Material examined: collecting data.
