# Supplementary material for: Comparative Morphology of Wax Gland Heads in Adult Dustywings (Insecta: Neuroptera: Coniopterygidae)
Source: Insects. 2023 Jul 20;14(7):650. doi: 10.3390/insects14070650 (PMC10380582; doi:10.3390/insects14070650)
Supplement: Supplementary file 1 [file insects-14-00650-s001.zip › insects-2355327-supplementary.pdf]

Comparative Morphology of Wax Gland Heads in Adult Dustywings (Insecta: Neuroptera: Coniopterygidae)

Min Li [1], John D. Oswald [2,*] and Zhiqi Liu [1,*]

[1] Department of Entomology, China Agricultural University, Beijing 100193, China; liminbetter@126.com

[2] Department of Entomology, Texas A&M University, College Station, TX 77843, USA

* Correspondence: j-oswald@tamu.edu (J.D.O.); liuzhiqi@cau.edu.cn (Z.L.)

Table S1. Material examined: collecting data.

Subfamily: Tribe<br>***Genus (Subgenus)***<br>***Species***	**Province: Locality**	**Latitude Longitude** (decimal degrees)	**Date**
**Aleuropteryginae: Aleuropterygini**			
Aleuropteryx sinica Liu *et* Yang, 2003	Sichuan: Panzhihua	25.01°N 98.48°E	2019.04.03
Heteroconis terminalis Banks, 1913	Hainan: Danzhou	19.52°N 109.58°E	1974.12.07
" "	Yunnan: Puer	22.02°N 99.09°E	2019.03.30
**Aleuropteryginae: Coniocompsini**			
Coniocompsa longqishana Yang & Liu, 1993	Fujian: Jiangle	26.28°N 117.13°E	1991.10.10
**Aleuropteryginae: Fontenelleini**			
Cryptoscenea orientalis Yang & Liu, 1993	Guangxi: Baise	23.90°N 106.62°E	2019.04.06
" "	Guizhou: Luodian	25.04°N 160.23°E	1987.08.26
Spiloconis sexguttata Enderlein, 1907	Gansu: Longnan	34.35°N 104.21°E	2018.07.12
**Coniopteryginae: Coniopterygini**			
Coniopteryx (Coniopteryx)			
Coniopteryx aspoecki Kis, 1967	Shandong: Jinan	36.40°N 117.00°E	2020.10.31
Coniopteryx choui Liu & Yang, 1998	Jiangsu: Yangzhou	32.24°N 119.26°E	2020.11.02
Coniopteryx compressa Yang & Liu, 1999	Guangxi: Nanning	22.48°N 108.22°E	2020.11.13
Coniopteryx unispinalis Liu & Yang, 1994	Guangdong: Guangzhou	23.08°N 113.14°E	2019.11.10
Coniopteryx protrufrons Yang & Liu, 1999	Fujian: Fuzhou	25.15°N 118.08°E	2020.11.08
Coniopteryx gibberosa Yang & Liu, 1994	Fujian: Fuzhou	25.15°N 118.08°E	2020.11.08
Coniopteryx bispinalis Liu & Yang, 1993	Yunnan: Kunming	25.20°N 120.45°E	2019.03.17
Coniopteryx praecisa Yang & Liu, 1994	Yunnan: Puer	22.02°N 99.09°E	2019.03.21
Coniopteryx alticola Sziráki, 2002	Yunnan: Yuxi	23.19°N 101.16°E	2019.03.19
Coniopteryx (Xeroconiopteryx)			
Coniopteryx qiongana Liu & Yang, 2002	Guangdong: Huizhou	23.08°N 114.41°E	2019.08.25
Coniopteryx unguigonarcuata Aspöck *et* Aspöck, 1968	Xinjiang: Wulumuqi	42.45°N 86.37°E	2019.08.22
Thecosemidalis yangi Liu, 1995	Xinjiang: Kashi	38.50°N 75.47°E	1979.09.01
**Coniopteryginae: Conwentziini**			
Conwentzia sinica Yang, 1974	Shandong: Jinan	36.40°N 117.00°E	2021.06.28
Conwentzia nietoi Monserrat, 1982	Yunnan: Dehong	24.18°N 97.80°E	2019.03.28
Conwentzia pineticola Enderlein, 1905	Gansu: Gannan	33.96°N 103.55°E	2017.07.13
Semidalis aleyrodiformis Stephens, 1836	Beijing: Haidian	39.53°N 116.03°E	2021.08.22
Semidalis unicornis Meinander, 1972	Guangdong: Huizhou	23.08°N 114.41°E	2019.08.26
Semidalis tibetana Zhao et al., 2021	Xizang: Linzhi	26.52°N 92.09°E	2019.06.04
Semidalis anchoroides Liu & Yang, 1993	Xizang: Linzhi	26.52°N 92.09°E	2019.06.02
Semidalis decipiens Roepke, 1916	Yunnan: Jinghong	21.27°N 100.25°E	2019.03.23
Semidalis bicornis Liu & Yang, 1993	Sichuan: Panzhihua	25.01°N 98.48°E	2019.04.04
Semidalis procurva Zhao et al., 2021	Yunnan: Ruili	24.02°N 97.86°E	2019.03.30

Semidalis daqingshana Liu & Yang, 1994	Yunnan: Kunming	25.20°N 120.45°E	2019.03.16
